# Subthalamic stimulation causally modulates human voluntary decision-making to stay or go

**DOI:** 10.1038/s41531-024-00807-x

**Published:** 2024-11-02

**Authors:** Yichen Wang, Linbin Wang, Luis Manssuer, Yi-jie Zhao, Qiong Ding, Yixin Pan, Peng Huang, Dianyou Li, Valerie Voon

**Affiliations:** 1 Institute of Science and Technology for Brain-Inspired Intelligence (ISTBI), Fudan University, Shanghai, 200433 China; 2 Department of Psychiatry, Addenbrookes Hospital, University of Cambridge, Cambridge, CB2 0QQ United Kingdom; 3 Clinical Research Center for Mental Disorders, Shanghai Pudong New Area Mental Health Center, School of Medicine, Tongji University, Shanghai, 200124 China; 4 Department of Neurosurgery, Ruijin Hospital, Shanghai Jiao Tong University School of Medicine, Shanghai, 200025 China

**Keywords:** Neurophysiology, Human behaviour

## Abstract

The voluntary nature of decision-making is fundamental to human behavior. The subthalamic nucleus is important in reactive decision-making, but its role in voluntary decision-making remains unclear. We recorded from deep brain stimulation subthalamic electrodes time-locked with acute stimulation using a Go/Nogo task to assess voluntary action and inaction. Beta oscillations during voluntary decision-making were temporally dissociated from motor function. Parkinson’s patients showed an inaction bias with high beta and intermediate physiological states. Stimulation reversed the inaction bias highlighting its causal nature, and shifting physiology closer to reactive choices. Depression was associated with higher alpha during Voluntary-Nogo characterized by inaction or inertial status quo maintenance whereas apathy had higher beta-gamma during voluntary action or impaired effortful initiation of action. Our findings suggest the human subthalamic nucleus causally contributes to voluntary decision-making, possibly through threshold gating or toggling mechanisms, with stimulation shifting towards voluntary action and suggest biomarkers as potential clinical predictors.

## Introduction

Voluntary decision-making involves intentional choices substantially driven by internal factors, similar to deciding a ‘Go’ or ‘Nogo’ action towards a warning yellow traffic light. This process is distinct from reactive decision-making, an automatic reaction primarily triggered by external stimuli, such as responding to a green or red traffic light. The voluntary nature of decision-making is fundamental to human behavior in navigating our diverse and dynamic environment^1^. Human imaging studies have identified a role for the cortico-basal ganglia circuit in voluntary decision-making^2^; however, these studies measure metabolic, not neural activity, thus offering indirect and correlational results^3^. Thus, direct, causal and precise evidence of the role of the basal ganglia in voluntary decision-making remains to be shown.

Deep brain stimulation (DBS) captures the electrophysiological characteristics of cognitive processes by recording local field potentials (LFPs) with both spatial and temporal precision via the implanted electrodes^4^. Despite the importance of voluntary decision-making, most DBS studies have focused on its reactive form. Using classical paradigms like Go/Nogo tasks and stop-signal tasks, the subthalamic nucleus (STN), found within the indirect pathway of the cortico-basal ganglia circuit and receiving prefrontal hyper-direct projections, has been proposed to be functionally engaged in reactive decision-making. Specifically, the Go response is classically associated with beta (13–30 Hz) desynchronization or a decrease in beta power along with an increase in gamma power, while the Nogo response is characterized by beta synchronization or an increase in beta power^5,6^. However, these tasks assess reactive choice and less is known how or if the STN is engaged in voluntary activity.

The ability to gate movement may also grant the STN the ability to regulate voluntary decision-making, as one recent empirical study extended the role of beta oscillations in the STN to proactive inhibition^7^. Nevertheless, conventional paradigms do not cover the concept of real-world voluntary decision-making to choose between action and inaction. There are often circumstances where no prepotent actions need to be restrained, whereas most tasks focusing on motor response inhibition commonly have greater Go relative to Nogo responses to enhance Go responding and motor response prepotency. Furthermore, existing studies also commonly neglect the voluntary nature of the Go component although this is more often studied. To bridge this gap, we introduce an alternative paradigm that offers equivalent voluntary choices between action and inaction, allowing participants to decide freely whether to execute an action or not. Voluntariness may also be relevant in disorders of clinical pathology. For instance, impaired motor initiation deficits in Parkinson’s disease may be relevant to bradykinesia and akinesia along with a potential role for cognitive initiation deficits in the common comorbidity of apathy and depressive symptoms may also reflect underlying deficits in voluntary action and inaction.

DBS further allows unique insight beyond correlative observations, allowing a precise understanding of causality by delivering electrical pulses to modulate functioning of the specific neural target^8^. Chronic high-frequency (e.g., 130 Hz) stimulation of the STN appears to restore voluntary control in patients with Parkinson’s disease as illustrated by the alleviation of motor symptoms. STN-DBS can also interfere with decision-making processes particularly in tasks with high cognitive demand, as demonstrated in adjusting decision threshold^9,10^, decreasing reaction time, and inducing impulsivity^10,11^. Cumulatively, this evidence suggests an extended role of the STN in voluntary decision-making, which necessitates significant cognitive resources compared to reactive decision-making, beyond its function of supporting the decision-leading motor response.

In the present study, we recruited patients with Parkinson’s disease who had undergone DBS of the STN and recorded physiological activity during a modified Go/Nogo task with equal trials of Go and Nogo to capture the nature of real-world decision-making. The patients either responded with ‘Go’ or ‘Nogo’ towards reactive cues, or made a free decision on whether to respond in intentional trials, while concurrent LFP within the STN was recorded. The neural characteristics between voluntary and reactive decision in both choices were directly compared. In addition to the well-established dissociation between Go and Nogo actions in beta oscillatory activity^5^, we expected an effect of voluntariness in toggling between choices^12^. Because human voluntariness is mediated by endogenous factors such as motivational states and mood, and that voluntary initiation may also be specifically relevant to such states, we further assessed physiological biomarkers relevant to the syndrome of apathy and depression commonly observed in Parkinson’s disease^13–15^. Moreover, we interrogated effective functioning of the STN by delivering brief subthalamic stimulation time-locked to the preparatory stage prior to the cue onset. Trial-by-trial analyses were performed to capture the causal link between the variation of STN activity and voluntary behavior. We expected that brief subthalamic stimulation prior to the decision-making stage would influence voluntary choices, which could be predicted as a function of STN oscillatory activity. Our findings may contribute to a broader understanding of the STN in higher-level cognitive function.

## Results

### Behavioral characteristics of the modified Go/Nogo task

**Fig. 1:**
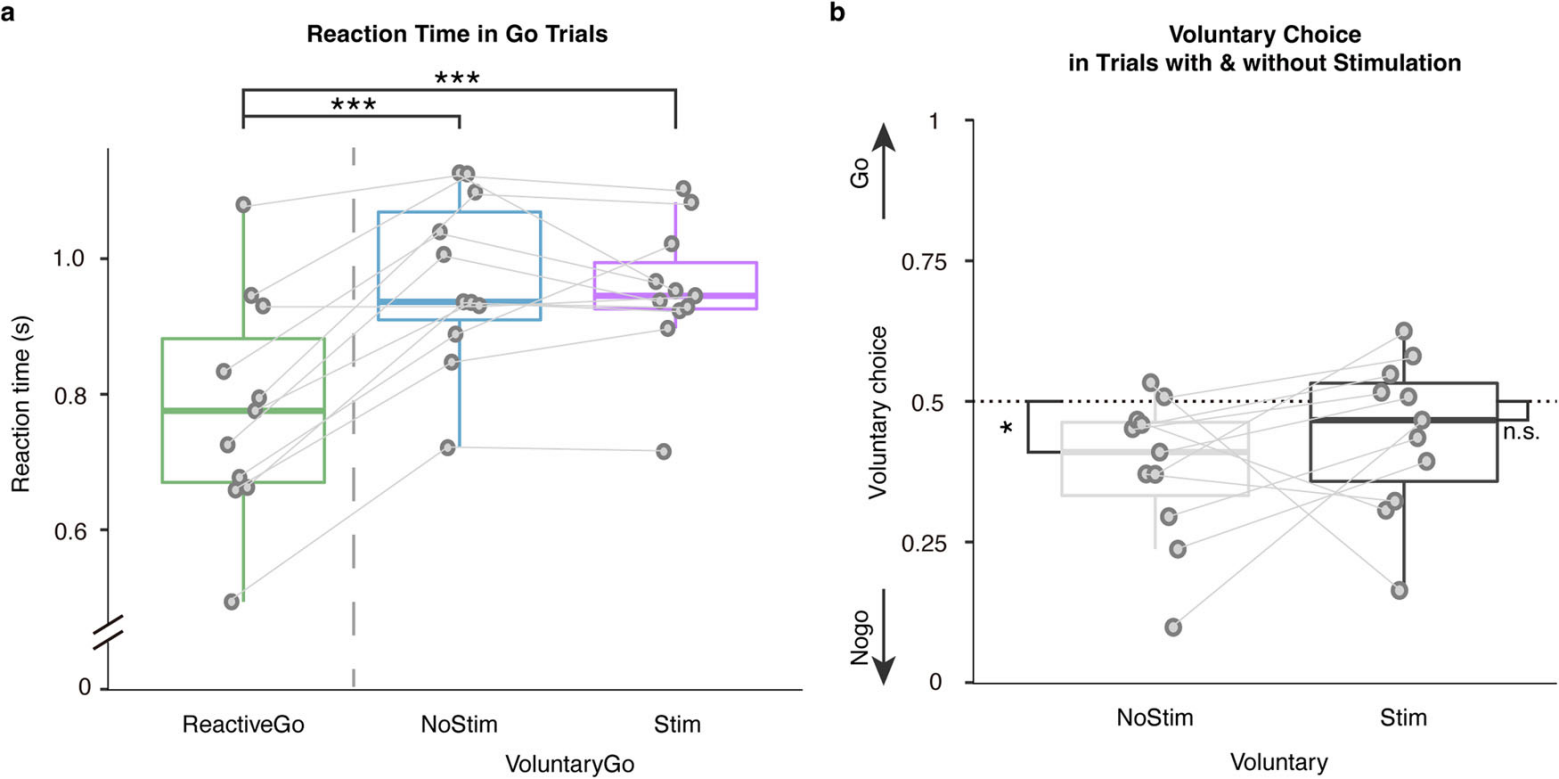
Behavioral characteristics of the modified Go/Nogo task. **a** Reaction times were compared between Go conditions, showing longer reaction times in Voluntary-Go both with and without subthalamic stimulation relative to the Reactive-Go condition. **b** Comparison of voluntary choice pattern between conditions with and without stimulation showed that subthalamic stimulation elicited more voluntary choices to ‘Go’. The box indicates the first and third quartile, the center bold line of the box indicates the median, and whisker lengths reflect the interquartile range multiplied by 1.5. The solid circles represent individual mean reaction times, and voluntary choice ratio, respectively. All p values in the paired t-tests were adjusted for multiple comparisons with Benjamini–Hochberg correction, ***p < 0.001, *p < 0.05. The black * symbols indicate a significant difference between corresponding conditions. The n.s. symbol indicates a non-significant result.

Reaction times were compared between Go conditions. Trials in which patients responded incorrectly, or responded faster than 300 ms were excluded from the analysis. Reaction times were significantly faster on Reactive-Go trials $(779.74\pm 163.39{ms})$, relative to Voluntary-Go $(968.64\pm 125.93{ms},t\left(10\right)=6.69,P < 0.001,{\rm{Cohen}}^{\prime} {\rm{s}}\,d=2.02)$, and also to Voluntary-Go with stimulation $\left(952.26\pm 102.85{ms},t\left(10\right)=-4.32,P=0.0015,{\rm{Cohen}}^{\prime} {\rm{s}}\,d=1.30\right.$; Fig. 1a).

Furthermore, we sought the effect of subthalamic stimulation on voluntary decision-making. Voluntary choices in PD patients were significantly biased to Nogo where 50% represented equal Go and Nogo choices as instructed during the task $(0.62\pm 0.13,t\left(10\right)=3.03,P=0.013,{\rm{Cohen}}^{\prime} {\rm{s}}\,d=0.91)$. In contrast, this inaction bias was not observed in voluntary trials paired with stimulation $\left(0.56\pm 0.14,t\left(10\right)=1.40,P=0.19,{\rm{Cohen}}^{\prime} {\rm{s}}\,d=0.42\right.$; Fig. 1b). As further shown in cross tabulation, subthalamic stimulation was associated with more voluntary choices to Go $\left({\chi }^{2}\left(1,N=1284\right)=6.51,P=0.011\right.$; Fig. 1c). Thus, critically, PD patients showed a baseline inaction bias which stimulation reversed facilitating voluntary action.

The above behavioral results remained after including patients whose DBS electrode for LFP recording was not well-localized in the STN (Supplementary Fig. 1).

### STN beta oscillations supports voluntariness in decision-making

**Fig. 2:**
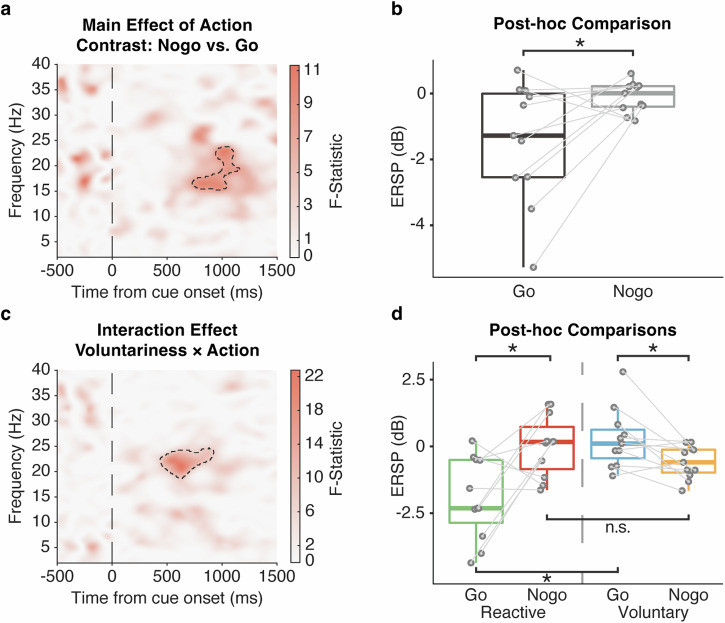
Time-frequency decomposition of the modified Go/Nogo task. Stimulus-locked time-frequency statistical result of ERSPs comparing the factors of voluntariness (Reactive, Voluntary), action (Go, Nogo) and their interaction during the decision phase controlled for UPDRS-III as a covariate of no interest. **a** Main effect of action (Comparing Nogo and Go, F-statistic), with (**b**) post-hoc paired t-test showing that the action of Go elicited a decrement in the STN ERSPs relative to Nogo. **c** Interaction effect of voluntariness and action (F-statistic), with further (**d**) post-hoc paired t-tests showing the dissociated effect of voluntariness on Go and Nogo trials, as well as the neural characteristics between Go and Nogo action differentiated in Voluntary and Reactive conditions. All the time-frequency plots were time-locked to the cue onset (time = 0). Significant cluster findings in the time-frequency plots are shown in black dashed lines. The post-hoc paired t-tests were performed with individual ERSPs averaged within the significant cluster, as represented by the solid circles. Boxplot box indicates the first and third quartile, the center bold line of the box indicates the median, and whisker lengths reflect the interquartile range multiplied by 1.5. All the *p* values in the post-hoc t-tests were adjusted for multiple comparisons with Benjamini–Hochberg correction, *p < 0.05. The black * symbols indicate a significant difference between corresponding conditions. The n.s. symbol indicates a non-significant result.

To investigate the functional engagement of the STN in voluntary decision-making, a full factorial model was conducted to assess the within-subject main effects of voluntariness (Reactive, Voluntary), action (Go, Nogo) and their interaction effect, with the UPDRS-III score being controlled as a covariate of no interest. As expected, a main effect of action was observed in STN beta (15–25 Hz) ERSP around 1000 ms post-stimulus $\left(F\left({1,39}\right)=10.62,Z=2.83,P < 0.001,{FWE}\right.$; Fig. 2a), with the post-hoc t-test further confirming beta power suppression on Go relative to Nogo trials (*t*(10) = −2.54, *P* = 0.029, Cohen's *d* = 0.76; Fig. 2b). Importantly, an interaction effect of voluntariness and action was observed in STN beta (20–25 Hz) ERSP earlier at 500 ms post-stimulus $\left(F\left({1,39}\right)=22.77,Z=4.05,P < 0.001,{FWE}\right.$; Fig. 2c): Voluntary-Go was characterized by an increase in the ERSP relative to Reactive-Go $(t\left(10\right)=3.91,P=0.011,{{\rm{Cohen}}}^{{\prime} }{\rm{s}}\,d=1.19)$, whereas no significant difference was observed in between Voluntary- and Reactive-Nogo $(t\left(10\right)=-1.61,P=0.12,{\rm{Cohen}}^{\prime} {\rm{s}}\,d=0.52)$. Notably, the ERSP was lower in Reactive-Go relative to Reactive-Nogo as expected $(t\left(10\right)=-3.14,P=0.021,{\rm{Cohen}}^{\prime} {\rm{s}}\,d=0.95)$, but this pattern appeared to be reversed between Voluntary-Go and Nogo $\left(t\left(10\right)=2.69,P=0.030,{\rm{Cohen}}^{\prime} {\rm{s}}\,d=0.81\right.$; Fig. 2d). The interaction effect remained after further controlling for apathy $(F\left({1,38}\right)=25.11,Z=4.21,P < 0.001,{FWE})$, and depression $(F\left({1,38}\right)=26.45,Z=4.30,P < 0.001,{FWE})$ severity. We noted that this interaction effect occurred considerably earlier relative to the main effect of action around 1000 ms.

To further differentiate the motor-related effect from the interaction effect, we first performed a linear regression between the within-cluster ERSPs and individual mean reaction times in the Go trials, and performed the post-hoc t-test again with the residuals. The neural characteristics observed in Voluntary-Go relative to Reactive-Go remained $\left(t\left(10\right)=4.84,P < 0.001,{\rm{Cohen}}^{\prime} {\rm{s}}\,d=1.50\right.$; Supplementary Fig. 2). Moreover, as the interaction effect occurred earlier than 1000 ms, we further excluded Voluntary-Go trials with reaction time less than 1000 ms, and performed the analysis of variance in the same SPM model. Again, we observed the interaction effect from a similar beta cluster $\left(F\left({1,39}\right)=22.35,Z=4.02,P < 0.001,{FWE}\right.$; Supplementary Fig. 3). These results suggested a non-motor, but voluntariness-related STN beta band oscillatory activity during decision-making driven by a lack of beta desynchronization with voluntary action similar to reactive inaction. Instead, we observed a reversed relationship between this voluntariness-related STN beta and Go-Nogo in the voluntary domain, with greater beta activity to Voluntary-Go versus Nogo.

### STN reflects clinical characteristics relevant to voluntary decision-making

**Fig. 3:**
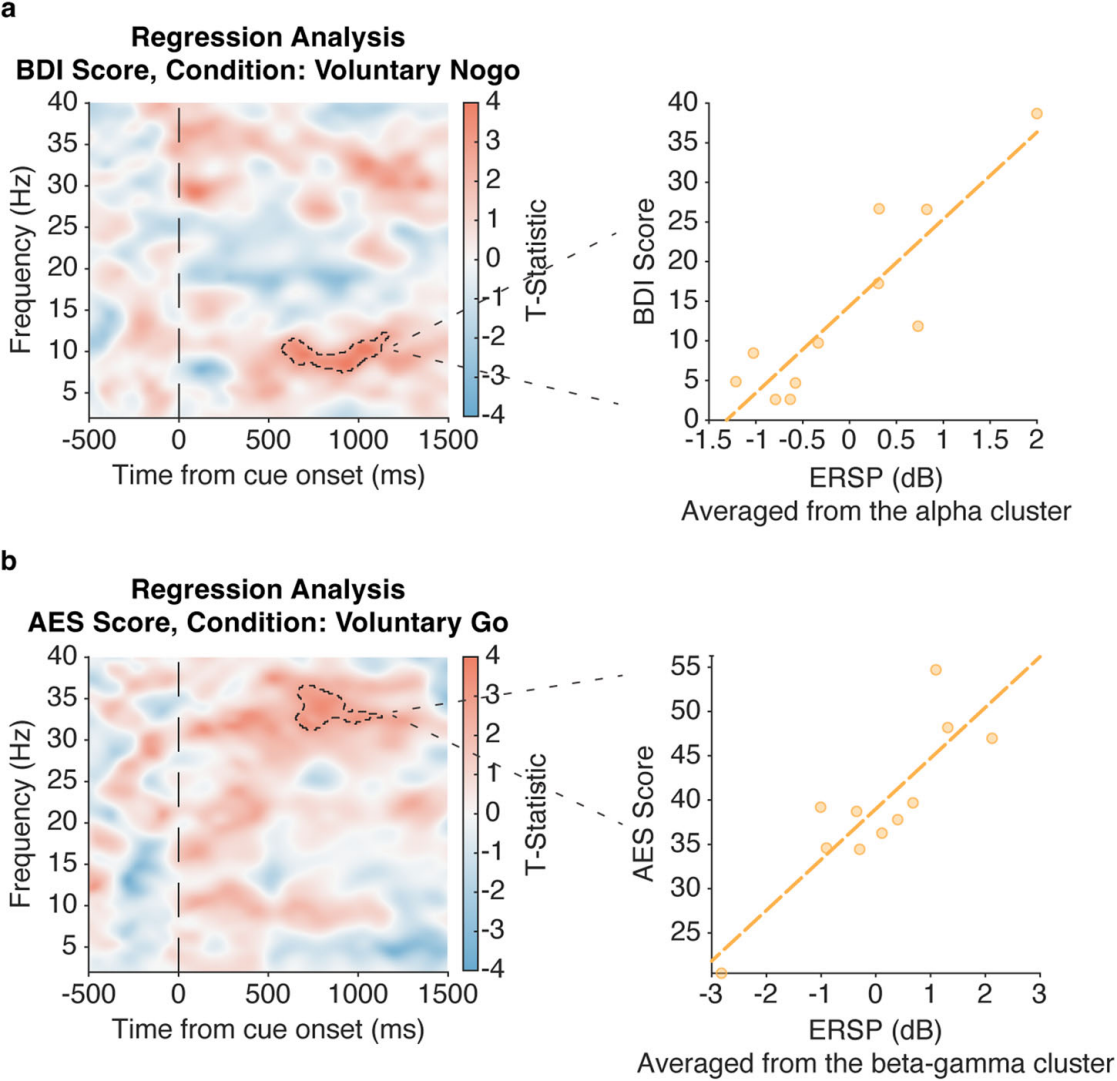
Electrophysiological correlate of apathy and depression in voluntary decision-making. Exploratory analysis of the stimulus-locked time-frequency power plot of within subthalamic (STN) spectral perturbation tested for regressors of clinical measures, controlled for UPDRS-III as a covariate of no interest. **a** Regression time-frequency plot (F-statistic) of the Voluntary-Nogo condition shows a positive relation between STN alpha (8–12 Hz) power and the Beck Depression Inventory (BDI)-II score, as visualized by scattergrams with individual ERSPs averaged within the identified cluster and BDI-II scores. **b** Similar time-frequency regression plot of the Voluntary-Go condition also shows a positive relation between STN beta-gamma (30–37 Hz) power and the Apathy Evaluation Scale (AES) score, as visualized by scattergrams with individual ERSPs averaged within the identified cluster and AES scores, further controlled for BDI-II score and reaction time. All regression plots were time-locked to the cue onset (time = 0). Significant cluster findings in the time-frequency plots are shown in black dashed lines. The scattergrams and fitted lines illustrate the relationship between individual ERSPs averaged within the significant cluster highlighted in the regression plots and clinical scores, for visualization purpose only.

On an exploratory basis, we then sought neural characteristics of apathy and depression severity, measured by AES and BDI-II respectively, within neural activity during voluntary decision-making (Voluntary-Go and Nogo). The time-frequency regression analyses showed that higher scores on the BDI-II, which indicates greater depression severity, were associated with stronger alpha activity (8–12 Hz, *t*(17) = 4.34, *Z* = 3.51, *P* = 0.0012, *FWE*; Fig. 3a) in the Voluntary-Nogo condition. We also observed an association between greater scores on the AES, which indicates lower apathy severity, and stronger beta-gamma activity (30–37 Hz, *t*(14) = 3.56, *Z* = 2.95, *P* = 0.0020, *FWE*; Fig. 3b) in the Voluntary-Go condition, controlled for reaction time and BDI-II scores. There were no associations observed between the neural activity in other conditions and AES or BDI-II scores (no cluster survived after cluster-level correction). Thus, depression and apathy were dissociated with greater depression associated with higher alpha power with voluntary inaction, and greater apathy associated with lower beta-gamma power with voluntary action.

### Subthalamic stimulation causally modulates voluntary decision-making mediated by beta oscillations

Subsequently, we investigated whether and how brief 500 ms right STN stimulation delivered at 1000 ms prior to the decision cue onset influenced voluntary decision-making. No complications or adverse events were reported by any patient. By comparing voluntary trials with and without stimulation, we assessed the causal nature of STN-DBS associated with voluntary decision-making, and its relationship with beta oscillations.

Since our analyses focused on the signal change relative to the pre-trial period, we first compared the power spectrum in the beta band (13–30 Hz), within the period from −500 ms to 0 ms, between trials without and with the stimulation. Indeed, the stimulation did not lead to significant modulation to the pre-trial beta power $(t\left(10\right)=0.91,P=0.39,{\rm{Cohen}}^{\prime} {\rm{s}}\,d=0.27)$. This result remained true while extending the frequency band of the power spectrum analysis to 2–40 Hz (*t*(10) = −0.97, *P* = 0.35, Cohen′s *d* = 0.29). This ensured that the stimulation-induced modulation to the neural activity, as shown below, is specific to the voluntary task, and not biased by a general stimulation effect on pre-trial physiology.

**Fig. 4:**
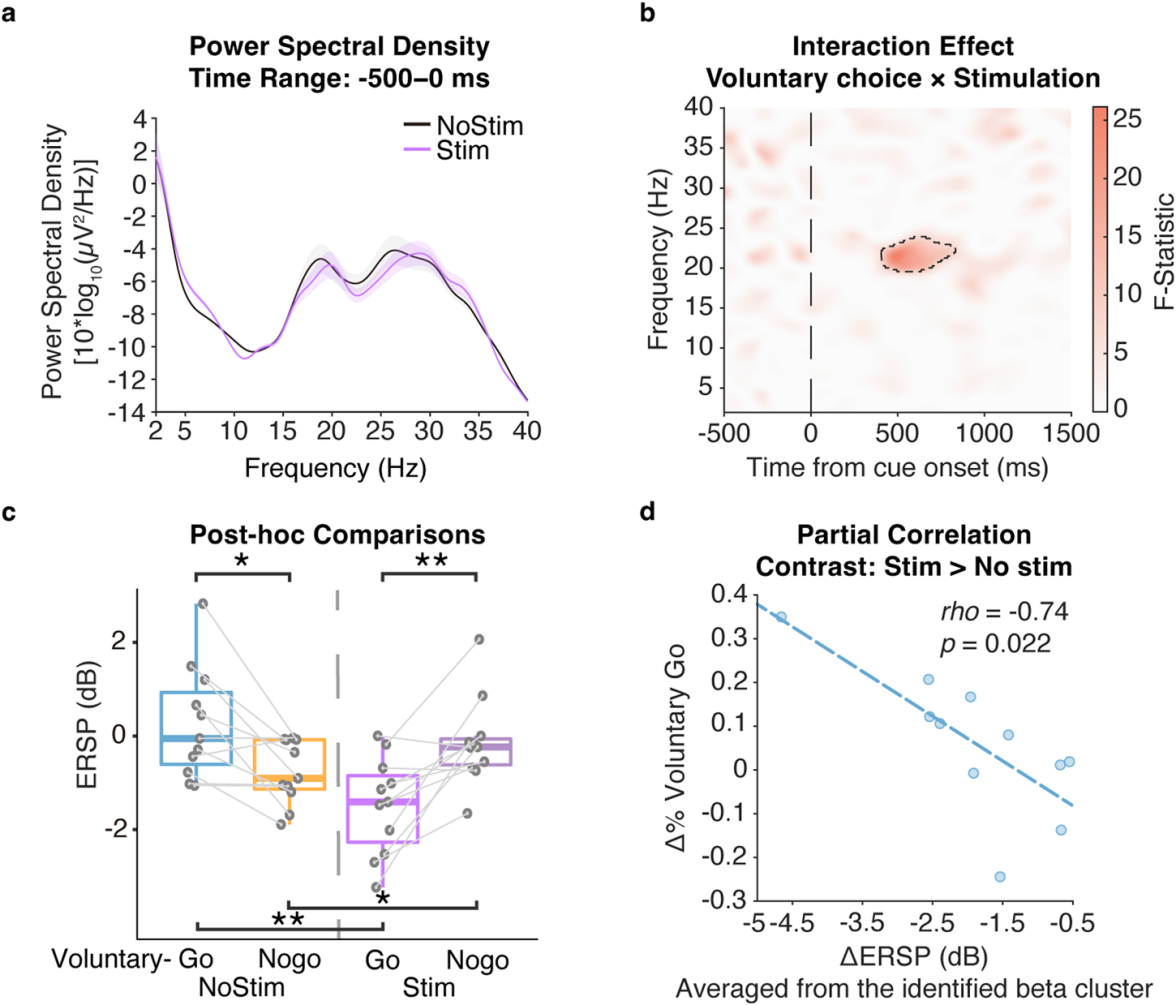
Electrophysiological and behavioral effect of acute subthalamic stimulation. Stimulus-locked time-frequency statistical result of ERSPs comparing the factors of voluntary choices (Voluntary-Go, Voluntary-Nogo), stimulation (Stimulation, No-stimulation) and their interaction during the decision phase controlled for UPDRS-III as a covariate of no interest. **a** Power spectral density showing the physiological effect of subthalamic stimulation on the pre-trial period. **b** Interaction effect of voluntary choice and stimulation (F-statistic), with further (**c**) post-hoc t-tests showing dissociated neural patterns of between Voluntary-Go and Voluntary-Nogo with and without subthalamic stimulation. **d** Partial correlation controlled for UPDRS-III score and reaction time shows that stimulation-induced decrement of beta power is associated with the increment of voluntary choice to ‘Go’, relative to the condition with no stimulation. Power spectra were averaged from 500 ms prior to the cue onset to the cue onset, and averaged for the conditions with subthalamic stimulation (purple) and with no stimulation (blue). Shading areas indicate standard error of mean. All the time-frequency plots were time-locked to the cue onset (time = 0). Significant clusters in the time-frequency plots are shown in black dashed line. The post-hoc paired t-tests were performed with individual ERSPs averaged within the significant cluster, as represented by the solid circles. Boxplot box indicates the first and third quartile, the center bold line of the box indicates the median, and whisker lengths reflect the interquartile range multiplied by 1.5. All the *p* values in the post-hoc t-tests were adjusted for multiple comparisons with Benjamini–Hochberg correction, *p < 0.05, **p < 0.01. The black * symbols indicate a significant difference between corresponding conditions. The scattergrams and fitted lines illustrate the correlation between individual ERSPs averaged within the significant cluster highlighted in the time-frequency statistical plot and voluntary choice proportion in condition with subthalamic stimulation relative to no stimulation.

A full factorial model was conducted to assess the within-subject main effects of stimulation on voluntary decision-making, comparing the physiology of voluntary choice (Voluntary-Go, Voluntary-Nogo), stimulation (Stimulation, No-stimulation) and their interaction effect, with the UPDRS-III score being controlled as a covariate of no interest. We observed an interaction effect of voluntary choice and stimulation on the STN beta band (20–25 Hz) ERSP again around 500 ms post-stimulus $\left(F\left({1,39}\right)=26.15,Z=4.29,P=0.0016,{FWE}\right.$; Fig. 4a): Voluntary-Go with stimulation was characterized by a decrease in the ERSP relative to Voluntary-Go without stimulation $(t\left(10\right)=-4.55,P=0.0042,{\rm{Cohen}}^{\prime} {\rm{s}}\,d=1.37)$, while an opposite effect was observed in the neural activity between Voluntary-Nogo with and without stimulation $(t\left(10\right)=2.93,P=0.015,{\rm{Cohen}}^{\prime} {\rm{s}}\,d=0.88)$. Thus, we observed a similar neural pattern as above between Voluntary-Go and Nogo without stimulation $(t\left(10\right)=3.17,P=0.013,{\rm{Cohen}}^{\prime} {\rm{s}}\,d=0.96)$, whereas critically, subthalamic stimulation reversed this relationship to be more similar to the reactive-like pattern $(t\left(10\right)=-3.94,P=0.0056,{\rm{Cohen}}^{\prime} {\rm{s}}\,d=1.19)$.

We then assessed the modulation effect of subthalamic stimulation on voluntary behavior, potentially via the neural activity within the identified beta cluster. Indeed, the stimulation induced decrease of ERSP within the identified beta cluster was correlated with the increase of Voluntary-Go choice relative to the non-stimulation condition $\left(r=-0.63,P=0.037\right.$; Fig. 4c), but not in Nogo condition $(r=0.045,P=0.89)$. Similarly, to rule out a motor-related effect, individual reaction times were excluded from the within-cluster ERSPs for respective Voluntary-Go conditions as the residuals from linear regression. The neural characteristics observed in Voluntary-Go with stimulation relative to non-stimulation condition remained $\left(t\left(10\right)=-3.95,P < 0.001,{\rm{Cohen}}^{\prime} {\rm{s}}\,d=1.05\right.$; Supplementary Fig. 4). Furthermore, as the interaction effect occurred earlier than 1000 ms, we excluded the Voluntary-Go trials with reaction time less than 1000 ms, and performed the analysis of variance in the same SPM model. Again, a consistent interaction effect was identified within the identical beta cluster $\left(F\left({1,39}\right)=19.89,Z=3.82,P=0.0059,{FWE}\right.$; Supplementary Fig. 5). These results suggested a non-motor modulation of subthalamic stimulation influencing the voluntary decision-making process via influencing STN beta band oscillations. We noted that there were outliers in this correlation analysis, which could possibly mislead the result. To address this issue, we further conducted trial-by trial analyses as below.

To better illustrate how subthalamic stimulation modulates voluntary decision-making for both neural activity and behavior, we conducted trial-by-trial analyses to capture the inter-trial variation. We first construct an LMEM with Voluntary choice (Voluntary-Go vs. Voluntary-Nogo), Stimulation (Stimulation [Stim], No-stimulation [No-stim]) and their interaction as the fixed effects, and trials as the random effect predictors:

1 $$\begin{array}{l}{\rm{ERSP}} \sim 1+{\rm{Voluntary}}\; {\rm{choice}}+{\rm{Stimulation}}+{\rm{Voluntary}}\; {\rm{choice}}\\\times {\rm{Stimulation}}+\left(1|{\rm{Trial}}\; {\rm{ID}}\right)\end{array}$$

where the ERSP refers to the spectral perturbation relative to the baseline activity, averaged within the significant cluster, and the Trial ID refers to the trial number.

This model was used to validate the interaction findings above on the neural activity within the beta cluster identified above. As expected, we observed a significant interaction effect of voluntary choice and stimulation (Table 1; *t*(1280) = −3.44, *P* *<* 0.001). Post-hoc comparison showed that with subthalamic stimulation, the beta cluster power was significantly lower in Voluntary-Go trials relative to Voluntary-Nogo $(t\left(649\right)=-3.53,P=0.0018,{\rm{Cohen}}^{\prime} {\rm{s}}\,d=0.28)$. This might be attributed to the effect of the stimulation on Voluntary-Go, as it decreased the beta cluster power relative to the non-stimulation condition $(t\left(512\right)=-3.21,P=0.0028,{\rm{Cohen}}^{\prime} {\rm{s}}\,d=0.28)$.

**Table 1:** Linear mixed-effect model (1) result

Model variables	Estimate
(Intercept)	−2.37*** (−2.78, −1.96)
Voluntary choice_Voluntary-Go	0.50 (−0.18, 1.18)
Stimulation_Stim	0.40 (−0.19, 0.99)
Voluntary choice_Voluntary-Go*Stimulation_Stim	−1.65*** (−2.59, −0.71)

The linear mixed-effect model at trial-level showed that the stimulation decreased the identified beta cluster ERSPs specifically in Voluntary-Go condition.

***p < 0.001.

Next, we asked how patients’ voluntary decision-making was related to the neural activity within the identified beta cluster and the stimulation condition. Here, we constructed a logistic binomial GLM model of the probability of Voluntary choice (p) as a function of the ERSP averaged within the identified beta cluster, Stimulation, and their interaction:

2 $${\rm{logit}}\left({\rm{p}}\right) \sim 1+{\rm{ERSP}}+{\rm{Stimulation}}+{\rm{ERSP}}\times {\rm{Stimulation}}$$

where logit(p) reflects the log-odds ratio that the voluntary choice of a trial is chosen to Go.

Consistent with the subject-wise findings above, we observed a significant interaction effect of the beta cluster activity and stimulation condition (Table 2, *t*(1280) = −3.46, *P* < 0.001). Thus, we segregated the data into two groups with different stimulation conditions, and conducted the GLM model respectively. For the model with stimulation, we observed a significant effect of the beta cluster activity $(t\left(649\right)=-3.47,P < 0.001)$. Specifically, as the beta cluster power decreased, patients’ probability of voluntary choice to Go increased. In contrast, this was not true in the model without stimulation $(t\left(631\right)=1.41,P=0.16)$. Therefore, right STN stimulation reversed the relationship between this early voluntariness-related beta and Voluntary Go-Nogo towards a pattern more similar to reactive choices.

**Table 2:** Linear logistic regression model (2) result

Model variables	Estimate
(Intercept)	−0.50*** (−0.68, −0.32)
ERSP	0.027 (−0.01, 0.065)
Stimulation_Stim	0.062 (−0.20, 0.32)
ERSP*Stimulation_Stim	−0.095*** (−0.15, −0.043)

The linear logistic regression model at trial-level showed that the stimulation, together with the identified beta cluster ERSPs change induced by stimulation related to a larger probability of voluntary choice to go.

***p < 0.001.

## Discussion

The function of the STN is well-established in movement facilitation and inhibition^16,17^, but less is known about its engagement in the decision-making process that determines the movement, especially when the decision is fully driven by one’s own volition. The present study examined the electrophysiological role of the STN in voluntary decision-making, using a modified Go/Nogo task with direct recordings via DBS electrodes well-localized within the STN. PD patients showed an inaction bias in voluntary choices, consistent with heightened pathological beta activity. We demonstrate that STN activity, as reflected by oscillations in the beta band, can encode both voluntary decision-making and movement, but in temporally separate time windows and statistically independent from each other. As expected, consistent with previous findings of Go/Nogo tasks testing motor responding and inhibition^5,18^, we observed a decrease in beta power and an increase in beta power at the time of the response. Moreover, the interaction effect occurred earlier at ~500 ms with a decrease in beta power with Reactive-Go (versus Voluntary-Go) and a decrease in beta power with Voluntary-Nogo (versus Reactive-Nogo). Whereas Reactive-Go and Nogo exhibited the same expected relationship with beta (lower activity to Reactive-Go), this relationship was reversed with Voluntary-Go and Nogo (lower activity to Voluntary-Nogo). In the following discussion we focus on this earlier voluntariness-related beta interaction which we believe is indicative potentially of the decision choice to act or not act.

We further demonstrate the mechanistic and causal importance of the STN by applying time-locked stimulation to the STN. Critically, brief early right sided subthalamic stimulation for 500 ms increased the proportion of individual voluntary choices to Go, shifting away from inaction, whilst simultaneously augmenting the previously-identified task-dependent drop in beta power. The implemented stimulation shifted the inverse relationship between Voluntary-Go and Nogo to a pattern consistent with reactive choices. The observed association between the behavioral and electrophysiological changes elicited by stimulation indicates a potentially mediating relationship between voluntary decision-making and STN beta oscillations. These results were further confirmed by trail-by-trial analyses considering trial-wise variation in the STN beta power.

How does the STN support the process of voluntary decision-making? Our findings can be potentially explained by a threshold effect or the effects of toggling between choices. Our early beta activity findings are consistent with the proposed theory that STN activity encodes the content set in service of the current task^19–22^, and gates the decision via implementing a task-specific threshold^10,22–26^. For instance, Patel et al. using single-neuron recordings demonstrated that STN activity encodes the upcoming financial decision within a brief temporal window (~500 ms) prior to the manifestation of the choice^19^. Similarly, we demonstrated that the voluntariness-related STN beta activity during this early decision phase encoded two alternative choices to Go or Stay between which the participants toggled during voluntary choices. This subsequently led ~500 ms later to an ultimate response depending on whether the neural characteristics tended to be Go or Nogo.

The higher beta activity during this decision phase in Voluntary Go is similar to higher beta activity during Reactive Nogo, consistent with our observed inaction bias in PD patients and likely related to underlying pathological beta activity. The decrease in beta activity with Voluntary Nogo or the choice not to act, rather than being inertial and inactive, appears to be an active intermediate state characterized by toggling between action and inaction. This finding is also potentially compatible with Logan’s Horse Race model of Go and Nogo decisions competing with the executed response enacted on crossing the threshold^27^. With STN stimulation, the decrement of beta oscillatory power in the STN DBS condition correlating with the increment of the number of Go responses suggests a potential role for early beta power in gating the decision via a threshold effect. Thus, this early STN beta power might play a potentially causal role in representing or gating the decision threshold.

A neural pattern of consistent toggling during voluntary decision-making has been observed in cortical activity using electroencephalogram, in which the amplitude of frontal-medial N2 during intentional choices lie between their reactive forms^12,28^. The mesial frontal cortex, including anterior cingulate cortex (ACC), and presupplementary motor area (pre-SMA), is implicated in intentional decisions^29–36^, along with other components of cognitive control and decision-making^37–40^. Using a multiple-alternative decision-making task with ultrahigh field magnetic resonance imaging, Keuken et al. ^22^ showed that STN activity increased with the choice alternatives, correlated with anterior cingulate cortex (ACC) activity. Thus, our early STN beta findings might similarly reflect toggling patterns observed during intentional choices in cortical surfaces consistent with the role of top-down modulation from the mesial frontal cortex to the STN in supporting voluntary decision making and voluntary actions. Future studies investigating this cortical-STN pathway by recording the STN and cortical activity simultaneously are indicated.

As expected, we observed longer reaction times during voluntary, relative to reactive decision-making. In line with previous findings using a similar experimental paradigm, participants commonly slow down during free choices^12,28,31,41^, supporting the notion that voluntary choices demonstrate a ‘freedom from immediacy’, and demand greater cognitive control compared with automatic and immediate stimulus-driven reactive choices^1^.

Patients with Parkinson’s disease showed a greater tendency towards inaction during voluntary choices in patients with Parkinson’s disease. Using similar modified Go/Nogo tasks, the mean rate at which healthy participants freely chose to act in intentional trials were consistently over 50% as instructed, even when the Nogo choice was assigned as prepotent^12,28,41^. Our observed behavioral characteristic of voluntary decision-making with greater inaction might serve as one of the endophenotypes of Parkinson’s disease, which could be attributed to an impairment of voluntary action^42,43^. As a result, in the context of free choices, patients with Parkinson’s disease are more likely to choose to do nothing, than to facilitate an effortful movement and may demonstrate intrinsic impairments in voluntary motor intention. This is consistent with the observation of excessive pathological beta oscillations or beta bursting activity in the cortico-basal ganglia loop in Parkinson’s disease^44^ as the possible underlying neural mechanism potentially interfering with oscillations in the same beta band supporting the process of voluntary decision-making^45^. As such, consistent with the proposed role of the decision threshold of the STN, STN activity in patients with Parkinson’s disease may be intrinsically biased towards the neural activity of Nogo or inaction, compared with the decreased beta power of the Go response.

Studies using lesions or inactivation of the parietal cortex or pre-supplementary motor area show an influence on the experience of agency^46,47^. Here we transiently stimulated the right STN for 500 ms using high frequency stimulation between 1000 ms to 500 ms before the cue to decide on voluntary action or inaction. The timing of this acute STN stimulation is similar to previous studies investigating brief high frequency STN stimulation influencing the Stroop conflict task presumably influencing the preparatory phase^48^. STN stimulation at high frequency is believed to transiently inactivate the STN thus acting similarly to a transient inactivation of the STN in assessing causal mechanisms.

Critically when we delivered brief 500 ms subthalamic stimulation, adapted from the therapeutic 130 Hz STN-DBS in patients with Parkinson’s disease^49,50^, 1000 ms prior to the cue onset, we show that the stimulation selectively increases voluntary decision-making towards the Go choice. This behavioral change was achieved via the modulation of Voluntary-Go-relevant beta oscillations, where transient stimulation decreased beta power towards Voluntary-Go like neural activity. These findings further consolidate our proposed notion of the STN in encoding and gating the optional choices.

The effect of acute 500 ms STN-DBS very early in the preparatory phase prior to the voluntary cue may be to transiently inhibit physiological activity and particularly the pathological increase in beta power linked to impairments in symptoms of Parkinson’s disease. Notably, we did not observe an immediate effect of STN stimulation on physiology 500 ms before the trial, but rather a task-specific effect of stimulation on subsequent choices. These findings highlight potentially a causal role of the STN in voluntary action.

How this may be implemented remains to be established. Acute STN-DBS during early motor preparation might transiently inhibit pathological activity to allow subsequently for the emergence of more normal voluntary pattern of activity (closer to 50% voluntary action similar to healthy controls) and for the transient decrease in beta activity to allow toggling between voluntary choices. STN-DBS might interfere with tonic inhibitory signaling for voluntary action either retrogradely from cortical regions or may allow the STN to be itself engaged in voluntary choice selection. Alternatively, brief STN stimulation might also normalize a pathologically impaired STN allowing it to respond in a voluntary manner. Thus, brief STN stimulation may potentially play a role in allowing the emergence of voluntary and neural choice behaviors.

Beyond the predominant motor symptoms, patients with Parkinson’s disease also experience a wide range of disabling mood and motivation disorders. Comorbid depression and apathy symptoms are common reported at 30%^51^ and 40%^52^ respectively. The distinction between apathy and depression in Parkinson’s disease is possible although is challenging and commonly highly comorbid^15,53^. From a mechanistic perspective, depression and apathy are both characterized by loss of motivation resulting in impaired behavioral activation and effort-based decision-making^54,55^. Here we sought to understand the neural characteristics of subjective apathy and depression severity in voluntary decision-making.

In line with previous findings highlighting the role of alpha oscillations in the STN in depressive or subjective emotional valence^8,56–59^, we show that the greater the severity of depression, the greater the alpha power during the decision-making stage of Voluntary-Nogo. As most patients with Parkinson’s disease with apathy have concomitant depression, with the prevalence dropping to less than 10% once depression and dementia are excluded^60^, we also included BDI-II scores as a covariate of no interest to focus on apathy specifically controlling for depressive symptoms. We show that a greater severity of apathy is associated with lower beta-gamma power during the decision-making stage of Voluntary-Go. These findings may be related to motivation, as studies have shown an increase of beta-gamma activity after rewards^61^.

Thus, our findings provide potential insights into the nature of voluntary decision-making underlying impairments in motivation of depression and apathy. Depression appears to implicate Voluntary-Nogo with high alpha power characterized by inaction or inertial maintenance of the status quo, whereas apathy appears to implicate voluntary action or impairments with low beta-gamma power in effortful initiation of actions. As such, although apathy from depression might both be characterized by motivational impairments, we show a physiological dissociation with volitional action and inaction respectively suggesting potentially different impairments in cognitive processes underlying these symptoms. These task-dependent neural markers for neuropsychiatric symptoms might contribute to developing closed-loop or neurofeedback therapy for mood and motivation symptoms in Parkinson’s disease.

This study is not without limitations. First, as patients with Parkinson’s disease are characterized by prominent excessive beta power in the STN^44^, the observed beta changes in the STN may be disease-related. However, we believe this may be relevant for baseline activity or manifest itself in the proportion of Voluntary-Nogo choices as we discuss, but as our task data involves within-subject controls for condition and baseline activity, we do not believe our findings are solely related to physiology in Parkinson’s disease. As the patients with Parkinson’s disease were also in the on-medication state^62^, and we further included subjective UPDRS-III score into our regression model as covariate of no interest, we consider our findings to likely represent task-related physiological changes. Another relevant issue is whether those results represent the decision phase relevant to intention or captured elements of motor function including preparation, initiation or execution. To address the issue, we leveraged the high temporal resolution of intracranial recordings, and the regression model to separate those two stages. As shown in the time-frequency analyses, consistent with the temporal characteristic of the well-established motor-induced beta desynchronization with the RT^5^, the time window of motor effect was around 1000 ms, while voluntariness was apparent earlier around 500 ms, which is before actual movement initiation (relative to mean RT). Considering the potential confounder of motor preparation with decision-making, we further included individual RTs^63^ into our later regression model (post-hoc t-test for the contrast of Go conditions) to fully regress out the motor component. Future studies might set separate periods for decision-making and motor response in the experimental task to dissociate those two stages directly. Moreover, although subthalamic stimulation exerted its effect on the beta cluster identified in the non-stimulation condition, which was considered as a non-motor component, we cannot rule out the possibility that subjective voluntary decision-making was modulated indirectly via the alleviation of motor symptoms^64^. However, considering that there is no significant difference between the RT in Voluntary-Go with and without stimulation, we do not believe the brief, time-locked stimulation resulted in prominent motor improvement. Furthermore, we included trial-wise fluctuations in beta power at baseline into our trial-by-trial model as a random effect, so that we separated the effect on task-relevant activity from the potential broader effect on beta power induced by the stimulation. Future studies should assess the motor symptoms after stimulation, and take the stimulation-related motor changes into account. Finally, we noted the significant male dominance within our participant group. Caution should be exercised when generalizing the above findings to women.

In conclusion, we provide direct electrophysiological evidence in human subjects demonstrating the essential causal role of the STN in voluntary decision-making. We also demonstrate task-dependent biomarkers of STN activity for non-motor symptoms related to Parkinson’s disease. Together, our findings extend current knowledge of the STN in voluntary decision-making, and also provide insights for advancing neuromodulation approaches for neurological and neuropsychiatric therapies.

## Methods

### Patients

This study was approved by the ethics committee of RuiJin Hospital, Shanghai Jiao Tong University School of Medicine (ethics approval number: 2019[220]), and written informed consent in accordance with the Declaration of Helsinki was obtained from each patient. Fifteen patients with Parkinson’s disease who had undergone STN-DBS surgery at RuiJin Hospital, Shanghai Jiao Tong University School of Medicine were initially recruited. Using LEAD-DBS, we further ensured that at least one DBS electrode was well-localized in the STN, resulting the exclusion of two patients with their DBS electrode localized outside the STN (distance to the STN > 1 mm). Furthermore, two other patients consistently made no response in the voluntary trials. Consequently, a total of eleven patients were included in the subsequent analyses. The detailed inclusion and exclusion criteria were reported in a previous study^57^.

Prior to the surgery, motor functioning was tested after a washout period approximately 12 h from their last routine dose of anti-parkinsonian medication, and one hour after a suprathreshold dose of levodopa (L-dopa). They were assessed by experienced neurologists using the motor subscale of the Unified Parkinson’s Disease Rating Scale (UPDRS-III). Patients were also assessed with the Montreal Cognitive Assessment (MoCA) for general cognitive function, the Apathy Evaluation Scale (AES) for apathy severity (here, illustrated in reverse with higher scores indicated lower apathy severity), and the Beck Depression Inventory (BDI)-II for depression severity. Details of the clinical characteristics and basic demographics of the patients are listed in Table 3.

**Table 3:** Patient demographic

Patient	Age(y)	Gender	Handedness	Education (y)	Disease duration (y)	Levodopa equivalent dose (mg)	H&Y stage OFF Medication	H&Y stage ON Medication	UPDRS-III OFF Medication	UPDRS-III ON Medication	MoCA	AES	BDI-II	Electrode model	Contact analyzed
1	73	Male	Rightness	9	11	750	2.5	2	40	27	24	12	41	Medtronic 3387S	L1 (L0)
2	65	Female	Rightness	13.5	10	937.5	4	3	59	40	20	31	26	Medtronic 3387S	L0 (L1)
3	57	Male	Rightness	23	10	963	3	2	41	23	26	39	7	Medtronic 3387S	L1 (L0)
4	34	Male	Rightness	10	20	600	4	3	96	41	26	49	11	SceneRay 1210-30/40	L1 (L0)
5	48	Male	Rightness	11	5	1105	4	2	59	10	28	30	2	PINS L302	L1 (L0)
6	50	Male	Rightness	5	8	437.5	3	3	46	20	22	54	4	Medtronic 3387S	L1 (L0)
7	47	Male	Rightness	12	10	1150	2.5	2	53	18	26	61	5	Medtronic 3387S	L0 (L1)
8	52	Male	Rightness	8	9	800	2.5	2	41	24	26	32	14	PINS L302	L0 (L1)
9	68	Male	Rightness	7	5	300	3	2.5	45	31	24	39	10	Medtronic 3387S	L0 (L1)
10	68	Male	Rightness	11	12	450	2	2	73	34	29	47	7	Medtronic 3387S	L0 (L1)
11	51	Male	Rightness	19	9	525	2.5	2	53	21	26	37	27	Medtronic 3387S	L0 (L1)

*UPDRS-III* Unified Parkinson’s Disease Rating Scale: Part III Score, *MoCA* Montreal Cognitive Assessment Score, *AES* Apathy Evaluation Scale Score, *BDI-II* Beck Depression Inventory Score, *Contact analyzed* columned refer to the reference contact.

The study was conducted during externalization, after the first 24 h post-surgery with the implantation of the electrodes and the implantable pulse generators, and before the second surgery with connection of the two devices. Patients were tested on medication at least 30 min after their regular medication dose. The impedance was below 10 kΩ.

### Modified Go/Nogo task

**Fig. 5:**
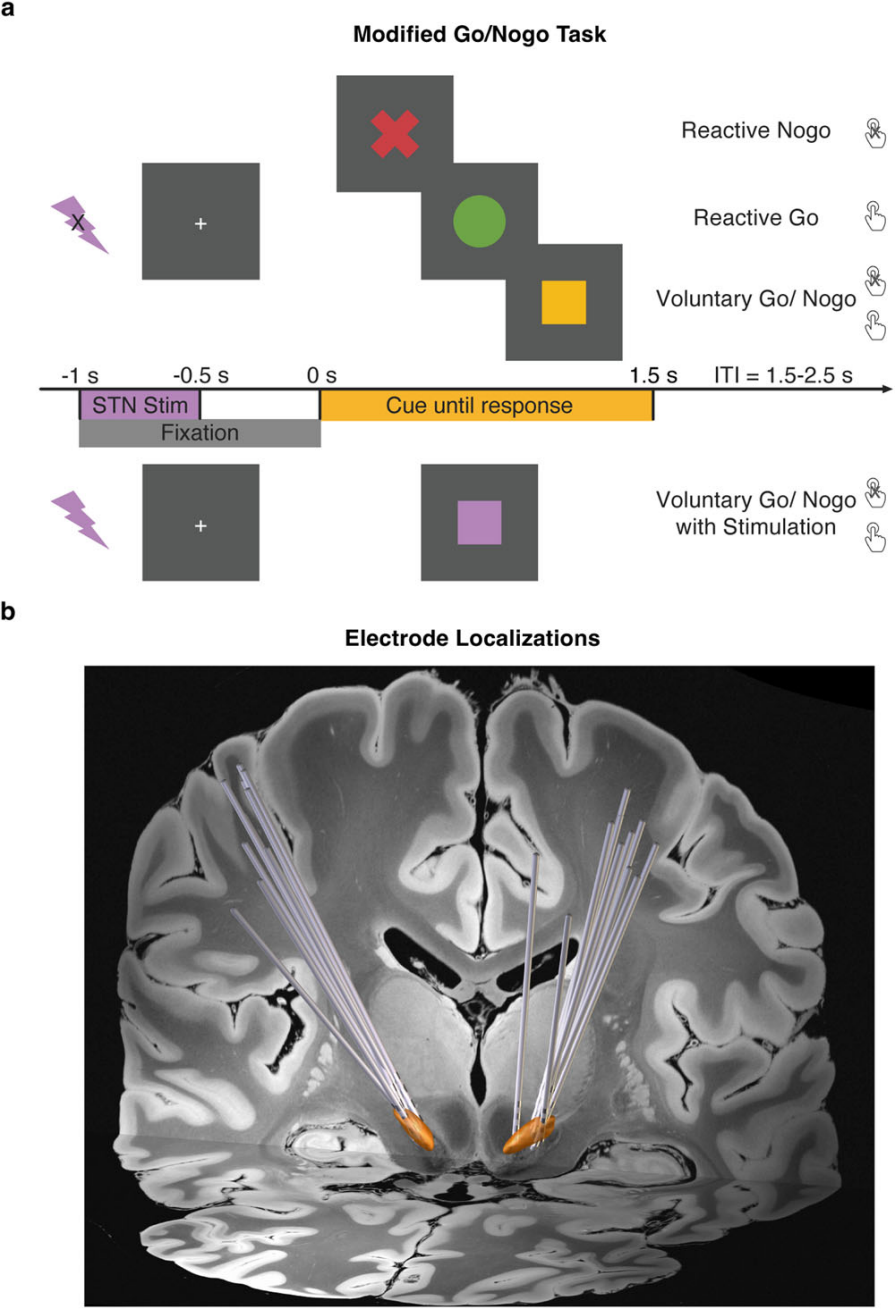
The modified Go/Nogo task paradigm. **a** The modified Go/Nogo task consisted of ‘reactive’, ‘voluntary’, and ‘voluntary with stimulation’ trials. In the ‘reactive’ trials, patients were instructed to either respond with a key press, or make no response, as indicated by the cue of a red cross or green circle respectively. In the ‘voluntary’ trials, patients made free choices between a key press response, or no response, as indicated by the yellow square cue. In the ‘voluntary with stimulation’ trials, patients made free choices as well. Right subthalamic stimulation at 130 Hz was delivered at 1000 ms prior to the cue onset, and lasted for 500 ms, which was indicated by a purple square. **b** Electrode reconstruction in Montreal Neurological Institute (MNI) space, with the coronal view displayed on a 7 T T1-weighted structural MRI template. The STN is highlighted in orange.

In the Modified Go/Nogo task (Fig. 5a), there were four conditions: Reactive-Go, Reactive-Nogo, and Voluntary-Go/Nogo and Voluntary-Go/Nogo with stimulation. The following describes the conditions without stimulation.

The cue indicated the action type: a green circle to press the space bar key with their left index finger as quickly as possible; a red ‘X’ to withhold from the key press, and a yellow square to decide whether to press the key or do no response voluntarily.

For the Voluntary-Go/Nogo condition, patients were told to aim for an equal proportion of button presses or no responses, to spontaneously choose at the time of the cue and not follow any specific patterns or use any strategies such as alternating or counting. The cue was presented for a maximum duration of 1.5 s, and disappeared after a button press, or remained on screen if no response was made. In both reactive conditions, patients were presented with a screen indicating they had reacted incorrectly if their response to the cued actions was incorrect. The intertrial interval, marked by a central fixation cross, was jittered between 1.5 to 2 s allowing sufficient time for a motor rebound effect. A total of 120 trials were conducted, with 30 trials in each of the two reactive conditions, and 60 trials in the Voluntary-Go/Nogo condition. Prior to the task, patients underwent 8 practice trials and were closely monitored to ensure they had fully understood the task instructions. If needed, patients were asked to repeat the task instructions during the practice session, and were reminded of the instructions. Both versions of this task were programmed and run in MATLAB using Psychtoolbox 3.0 functions^65^ and were displayed on an LG L1954 monitor, with a width/height of 380 × 300 mm and a resolution of 1280 × 1024 pixels.

A fourth condition of Voluntary-Go/Nogo was paired with time-locked stimulation (Fig. 5a). The stimulation was delivered, starting from 1000 ms prior to the cue onset, and lasting for 500 ms. The stimulation parameters are further described below in detail. The task condition was similar to the above Voluntary-Go/Nogo condition, but included stimulation and was cued by a purple square. In both conditions, patients decided to either press the space bar with the left index finger, or to not respond voluntarily. This condition had a total of 60 trials.

### Subthalamic simulation

The current pulses for the intermittent time-locked protocol were delivered using a pulse generator (SceneRay, model 1510, Suzhou, China) approved by the National Medical Products Administration, China. The precise time-based control was programmed within the experimental paradigm run on MATLAB.

The intermittent stimulation of the middle contacts of the right STN (Contact 1 cathode and Contact 2 anode in bipolar configuration) was delivered for 500 ms at 130 Hz, with a pulse width set at 90 μs. The intensity level was individualized for each patient (Table 3) using standard protocols for high frequency stimulation. The stimulation intensity was gradually increased by 1 mA at 130 Hz, until the patient reported side effects of paraesthesia. The intensity was then decreased in steps of 1 mA until no side effects were reported thus maintaining patient blinding. We further confirmed that the patients could not detect the onset, offset or presence or absence of stimulation. Complications and adverse events were monitored throughout the stimulation.

### Electrode implantation and localization

Patients underwent DBS implantation surgery in the following stages. The intended target coordinates of the STN were determined by merging the post-operative computed tomography (CT) and the pre-operative 3.0 Tesla magnetic resonance imaging (MRI) images using Surgiplan software (Elekta, Stockholm, Sweden). Quadripolar electrodes with four platinum-iridium contacts (Medtronic 3387S, Medtronic, Minneapolis, MN; PINS L302, PINS, Beijing, China; or SceneRay 1210-30/40, SceneRay, Suzhou, China) were stereotactically implanted into bilateral STN under general anesthesia. In the first surgery, patients underwent twist drill craniotomy and the DBS leads were externalized, which enabled recordings before the subcutaneous pulse generator was connected in the next upcoming surgery. The study was conducted during this externalization phase. The merged CT and MRI images were inspected separately by two experienced neurosurgeons to confirm STN targeting. The MRI images revealed no abnormal findings. No surgical complications were reported.

The electrode localization was reconstructed employing LEAD-DBS toolbox 2.5.3^66^. The preoperative T1-weighted anatomical MRI was co-registered to the postoperative CT using Advanced Normalization Tools^67^ (ANTs) and subcortical refine. A T2-weighted MRI was further co-registered to the T1-weighted MRI using statistical parametric mapping (SPM) to improve co-registration performance. The CT was then normalized to the MNI_ICBM_2009b_NLIN_ASYM brain using ANTs. Accuracy of co-registration and normalization was inspected visually. The electrode paths were determined automatically using the trac/core method, followed by manual adjustment^68^. Visualization of the electrode reconstruction was displayed on a background image using 7 T Ex vivo 100um Brain Atlas^69^, with the STN using DISTAL Atlas^70^ (Fig. 5b).

### LFP recording and preprocessing

The LFP data were recorded using a BrainAmp MR amplifier (Brain Products, Gilching, Germany) with a sample rate of 500 Hz, and a notch filter set at 50 Hz to remove power line noise interference. The left mastoid was used as the online reference electrode. The electro-oculogram (EOG) was recorded to confirm that blinks and saccades did not contaminate the data. The middle two contacts on the right electrode were used for stimulation, and therefore were not available for stimulation and recording simultaneously. Thus, we focus our analyses here on the left STN contacts. The LFP data were preprocessed and analyzed using MATLAB 2021a and EEGLAB functions^71^. The left LFP DBS contacts were re-referenced using a bipolar montage by subtracting adjacent contacts. This ensures that the obtained activity is restricted to the STN as volume conducted activity from distant brain regions and reference-related activity is canceled out^68,72,73^. The contact with the highest probability of being located in, or in contact with the STN was selected for analysis. Only contacts <1 mm from STN based on Lead DBS analysis were used in the analyses. The contact with the next highest probability of being in the STN, or closest to it, was used as the reference contact for bipolar re-reference.

The LFP data were then preprocessed following a standard procedure, including high-pass filtering at 1 Hz using ‘pop_eegfiltnew’, time-locked epoch into 3 s from 1 s prior to 2 s after the cue onset, baseline correction by subtracting the mean activity in the 1 s-fixation stage prior the cue onset, and visual inspection to remove epochs or electrodes contaminated with artefactual activity.

### Time-frequency decomposition

Time-frequency decomposition was performed using the ‘pop_newtimef’ function in EEGLAB^71^. The time period of interest was from 500 ms prior to 1500 ms after the cue onset. To account for the edge effects resulting from time-frequency transformation, the decomposition was performed on the extended epochs from 1000 ms before and 2000 ms after the cue onset. The extended part was then removed from subsequent analyses. The LFP data were convolved using Fast Fourier Transform and Hanning window tapering in the frequency range of 2–40 Hz. To visualize power changes across the frequency range, the mean baseline log power spectrum was subtracted from each spectral estimate, producing the event-related spectral perturbation (ERSP) results given in decibel (dB) relative to the baseline (period prior to the cue onset) activity. For each condition, the ERSPs were averaged across trials to create a time-frequency matrix per patient for the later statistical analyses. All trials in Go condition with reaction times below 300 ms were excluded from the analysis (total trials excluded across all patients: Reactive-Go: 4 trials, Voluntary-Go: 19 trials, Voluntary-Go with stimulation: 11 trials). Furthermore, spectral analyses were conducted to investigate the effect of subthalamic stimulation on the period immediately after the stimulation offset, but prior to the cue onset (from 500 ms before the cue onset to 0 ms) using the ‘pop_spectopo’ function in EEGLAB^71^ based on Welch’s method, given in µV2/Hz, and transformed by 10*log10 for visualization only.

### Statistical analyses

Behavioral measures of the bias in voluntary choice to Go or Nogo, and reaction times in Go conditions were tested using two-sided paired t-tests at subject level. The independence of the stimulation condition and the voluntary choice to Go or Nogo was tested using chi-square test in cross tabulation.

The power spectrum was averaged for conditions with and without stimulation per patient and compared using a two-sided paired t-test. The ERSPs were statistically analyzed using SPM-12 (Wellcome Department of Imaging Neuroscience, Institute of Neurology, London, UK), which affords greater sensitivity to detect significant effects than nonparametric methods^74^. The time-frequency matrices were converted into Neuroimaging Informatics Technology Initiative (NIfTI) images, and entered into a second-level full factorial design for the analysis of variance. As we tested for within-subject variables in repeated measurements, measurements from different levels of the variable were set as with equal variance, and dependent between levels. Moreover, we included the UPDRS-III scores in the OFF-medication state to control for the potential confound raised from motor symptoms in PD patients. We used a cluster-forming threshold of *p* = 0.01, but the cluster was considered significant if the random field theory (RFT) corrected cluster-level significance exceeded a Bonferroni–Holm correction for the number of comparisons^75^. If any significant cluster was identified, the spectral perturbation was averaged within the cluster per patient, and used for post-hoc comparisons with two-sided paired t-tests. The resulting *p* values were adjusted for multiple comparisons correction using Benjamini–Hochberg method. As psychiatric disease severity can also interfere with task-evoked brain reactivity^72^, clinical measures were mean centered and incorporated into the SPM model as covariates of no interest. For exploratory purposes to investigate potential biomarkers, clinical measures were also assessed as regressors to examine their associations with the voluntary spectral perturbation. Furthermore, to rule out the possibility that the observed effect was motor-related, individual mean reaction times in Go trials were incorporated as predictor variables for the averaged spectral perturbation within the cluster in linear regression model, and fitted with MATLAB function ‘regress’, where the residuals from model estimation were further used for post-hoc comparisons to control for motor-related effects.

To better illustrate how subthalamic stimulation interferes with voluntary decision-making, as well as the potential role of the STN oscillatory activity, trial-by-trial analyses were first conducted with a linear mixed-effects model (LMEM) using the ‘fitlme’ function in MATLAB. This method is advantageous over standard t-tests as it allows modeling trial-by-trial variation, and is not biased by the small differences in the number of trials between conditions. As the voluntary choice to Go or Nogo is binomial, it was further incorporated into a generalized linear model (GLM) with a logit link function using the ‘fitglm’ function in MATLAB. Details of the model construction were described further in the results. The significance of each factor in the LMEM and GLM was evaluated using t-statistics.

Effect sizes are reported as Cohen’s d for t-tests.

## Supplementary information

Supplemental Material

## Data Availability

All analyses were done in MATLAB using the open-source functions of EEGLAB toolbox and the SPM-12 toolbox. The data supporting the results of this study are available upon reasonable request from the corresponding authors.
