# Supplementary material for: Subthalamic stimulation causally modulates human voluntary decision-making to stay or go
Source: NPJ Parkinsons Dis. 2024 Nov 2;10:210. doi: 10.1038/s41531-024-00807-x (PMC11531569; doi:10.1038/s41531-024-00807-x)
Supplement: Supplementary file 1 — Supplemental Material [file 41531_2024_807_MOESM1_ESM.docx]

# Supplementary Material

**
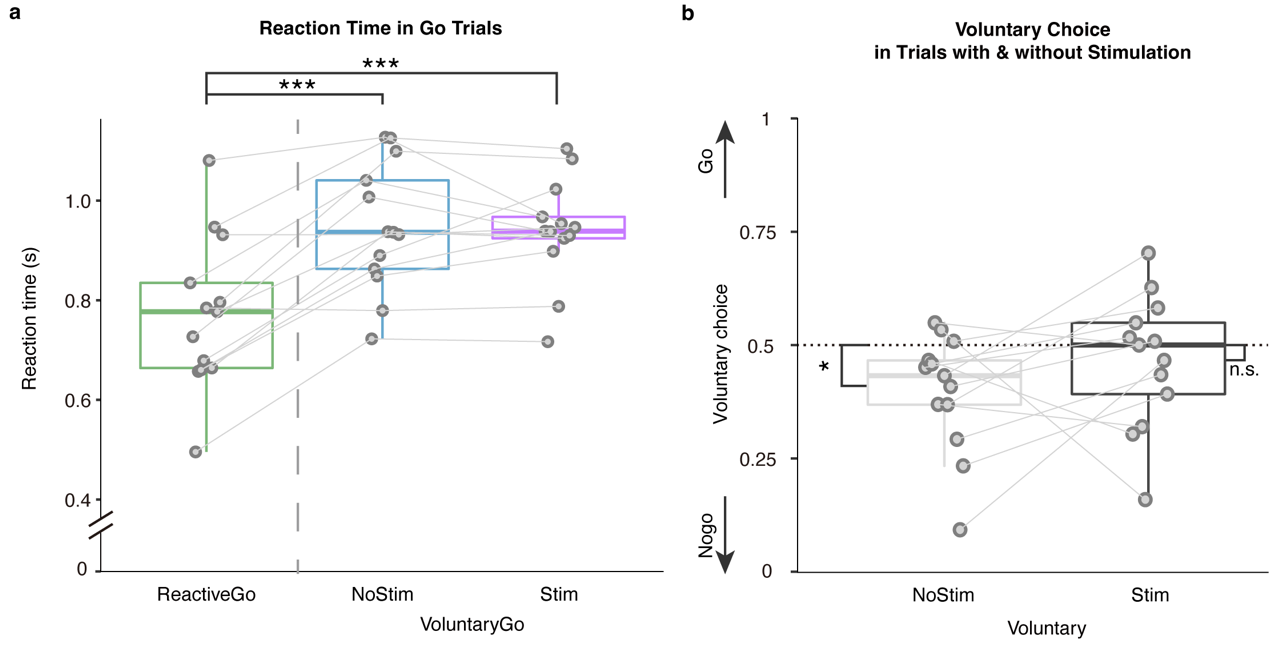
**

**Figure S1 Behavioral characteristics of the Modified Go/Nogo task with patients did not meet the DBS electrode criteria being included. (a)** The reaction times were compared between Go conditions, showing longer reaction time in Voluntary-Go both with and without subthalamic stimulation relative to Reactive-Go condition. **(b)** Comparison of voluntary choice pattern between condition with and without stimulation, showing the subthalamic stimulation elicited more voluntary choices to ‘Go’.

Boxplot box indicates the first and third quartile, the center bold line of the box indicates the median, and whisker lengths reflect the interquartile range multiplied by 1.5. The solid circles represent individual mean reaction times, and voluntary choice ratio, respectively. All the p values in the paired t-tests were adjusted for multiple comparisons with Benjamini-Hochberg correction, ***p < 0.001, *p < 0.05. The black * symbols indicate a significant difference between corresponding conditions. The n.s. symbol indicates a non-significant result instead.


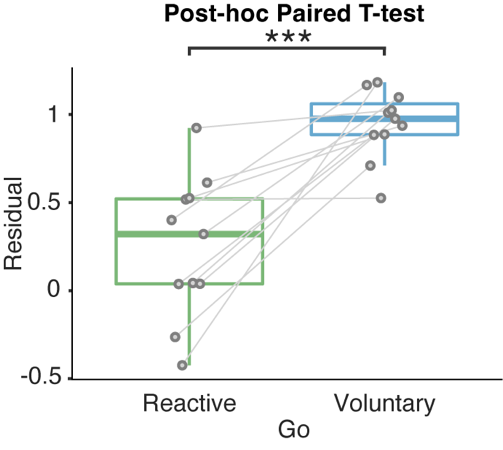


**Figure S2 Pairwise comparison between the ERSPs in Reactive- and Voluntary-Go with reaction time included as covariate of no interest.** The ERSPs were averaged within the identified cluster shown in Fig. 3c for Reactive- and Voluntary-Go condition respectively. Linear regression was performed between the averaged ERSPs and individual mean reaction time for each condition, and the resulting residuals were used for pairwise comparison as motor-related effect being controlled.

Boxplot box indicates the first and third quartile, the center bold line of the box indicates the median, and whisker lengths reflect the interquartile range multiplied by 1.5. The solid circles represent residuals per patient. The black * symbols indicate a significant difference between conditions, ***p < 0.001.


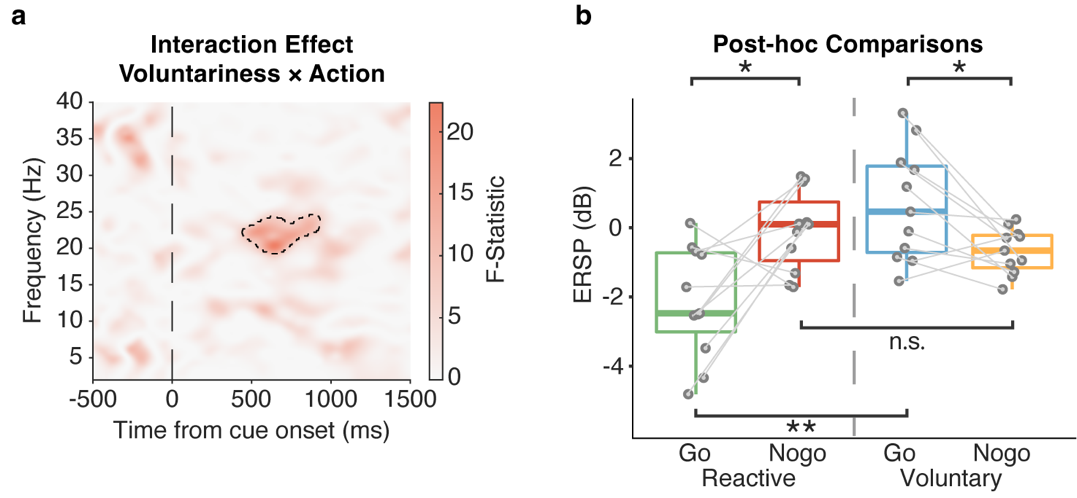


**Figure S3 Time-frequency decomposition of the modified Go/Nogo task with Voluntary-Go trials with reaction time less than 1000 ms being excluded.** Stimulus-locked time-frequency statistical result of ERSPs comparing the factors of voluntariness (Reactive, Voluntary), action (Go, Nogo) and their interaction during the decision phase controlled for UPDRS-III as a covariate of no interest. Here, only the interaction effect was presented. (**a**) Interaction effect of voluntariness and action (F-statistic), with further (**b**) post-hoc paired t-tests showing the dissociated effect of voluntariness on Go and Nogo trials, as well as the neural characteristics between Go and Nogo action differentiated in Voluntary and Reactive conditions. The results were similar to those shown in Fig. 3c, d.

All the time-frequency plots were time-locked to the cue onset (time = 0). Significant cluster findings in the time-frequency plots are shown in black dashed lines. The post-hoc paired t-tests were performed with individual ERSPs averaged within the significant cluster, as represented by the solid circles. Boxplot box indicates the first and third quartile, the center bold line of the box indicates the median, and whisker lengths reflect the interquartile range multiplied by 1.5. All the p values in the post-hoc t-tests were adjusted for multiple comparisons with Benjamini-Hochberg correction, *p < 0.05, **p < 0.01. The black * symbols indicate a significant difference between corresponding conditions. The n.s. symbol indicates a non-significant result instead.


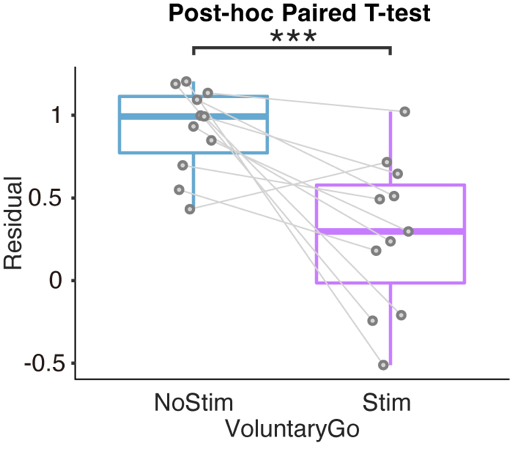


**Figure S4 Pairwise comparison between the ERSPs in Voluntary-Go with and without stimulation with reaction time included as covariate of no interest.** The ERSPs were averaged within the identified cluster shown in Fig. 5b for Voluntary-Go with and without stimulation respectively. Linear regression was performed between the averaged ERSPs and individual mean reaction time for each condition, and the resulting residuals were used for pairwise comparison as motor-related effect being controlled.

Boxplot box indicates the first and third quartile, the center bold line of the box indicates the median, and whisker lengths reflect the interquartile range multiplied by 1.5. The solid circles represent residuals per patient. The black * symbols indicate a significant difference between conditions, ***p < 0.001.


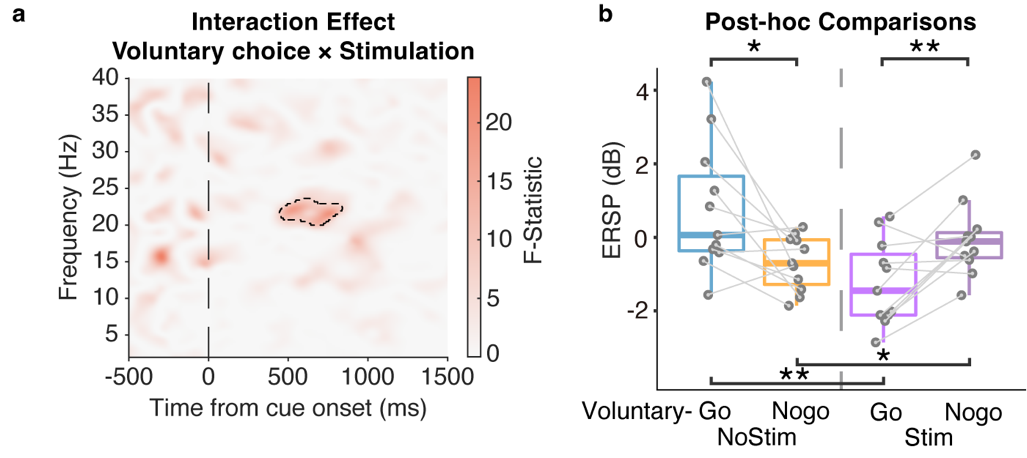


**Figure S5 Electrophysiological effect of acute subthalamic stimulation with Voluntary-Go trials with reaction time less than 1000 ms being excluded.** Stimulus-locked time-frequency statistical result of ERSPs comparing the factors of voluntary choices (Voluntary-Go, Voluntary-Nogo), stimulation (Stimulation, No-stimulation) and their interaction during the decision phase controlled for UPDRS-III as a covariate of no interest. **(a)** Interaction effect of voluntary choice and stimulation (F-statistic), with further **(b)** post-hoc t-tests showing dissociated neural pattern of between Voluntary-Go and Voluntary-Nogo with and without subthalamic stimulation. The results were similar to those shown in Fig. 5b, c.

All the time-frequency plots were time-locked to the cue onset (time = 0). Significant cluster finding in the time-frequency plots is shown in black dashed line. The post-hoc paired t-tests were performed with individual ERSPs averaged within the significant cluster, as represented by the solid circles. Boxplot box indicates the first and third quartile, the center bold line of the box indicates the median, and whisker lengths reflect the interquartile range multiplied by 1.5. All the p values in the post-hoc t-tests were adjusted for multiple comparisons with Benjamini-Hochberg correction, *p < 0.05, **p < 0.01. The black * symbols indicate a significant difference between corresponding conditions.
